# Isolated right ventricular Takotsubo cardiomyopathy in Guillain–Barré syndrome

**DOI:** 10.1186/s12872-022-02983-1

**Published:** 2022-12-07

**Authors:** Xiaojing Song, Cheng Chi, Junxian Song, Jihong Zhu

**Affiliations:** 1 Department of Emergency, Peking University People’s Hospital, Beijing, 100044 China; 2 Department of Cardiology, Peking University People’s Hospital, Beijing, 100044 China

**Keywords:** Isolated right ventricular Takotsubo cardiomyopathy, Guillain–Barré syndrome, Diagnosis, Electrocardiogram

## Abstract

**Background:**

Takotsubo cardiomyopathy can present itself in the most varied clinical forms, with extremely variable electrocardiogram anomalies and presence of comorbidities with a significant systemic commitment. Guillain–Barré Syndrome concomitant with isolated right ventricular Takotsubo cardiomyopathy is a rare entity. Here we present a patient with Guillain–Barré syndrome who had electrocardiogram abnormalities consistent with isolated right ventricular Takotsubo cardiomyopathy which have not been described in literature. This case report may prompt early identification of right ventricular involvement in neurological comorbidities, especially if the electrocardiogram is not frankly suggestive of an acute ischemic condition linked to coronary artery disease.

**Case presentation:**

A 37-year-old woman was misdiagnosed as acute coronary syndrome because of abnormally elevated troponin T level and electrocardiogram findings in the Emergency Department. Due to absence of any significant stenosis in the main coronary artery, the primary diagnosis was ruled out. Based on reanalysis of the ECG abnormalities, the patient was diagnosed as a case of isolated right ventricular Takotsubo cardiomyopathy in Guillain–Barré Syndrome. This case demonstrates the importance of electrocardiogram as a critical tool to identify isolated right ventricular Takotsubo cardiomyopathy in Guillain–Barré Syndrome. Indeed, in this case, the electrocardiogram abnormalities were distributed beyond the territory of a single coronary artery distribution.

**Conclusions:**

The described electrocardiogram findings of isolated right ventricular Takotsubo cardiomyopathy in Guillain–Barré Syndrome may facilitate identification of right ventricular involvement in neurological diseases.

**Supplementary Information:**

The online version contains supplementary material available at 10.1186/s12872-022-02983-1.

## Background

Guillain–Barré Syndrome (GBS) is an immune-mediated disorder which is caused by aberrant immune response against peripheral nerves via cross-reacting antibodies. GBS may be associated with cardiovascular complications [1], including arrhythmias, asystolia, blood pressure fluctuations, and the extremely rare condition called Takotsubo cardiomyopathy (TC). TC commonly affects the left ventricle. Right ventricular dysfunction may also occur but is exceedingly rare [2]. GBS concomitant with isolated right ventricular Takotsubo cardiomyopathy (RVTC) has never been reported before. Here we present a patient with GBS who had new ECG abnormalities consistent with isolated RVTC.

## Case presentation

**Fig. 1:**
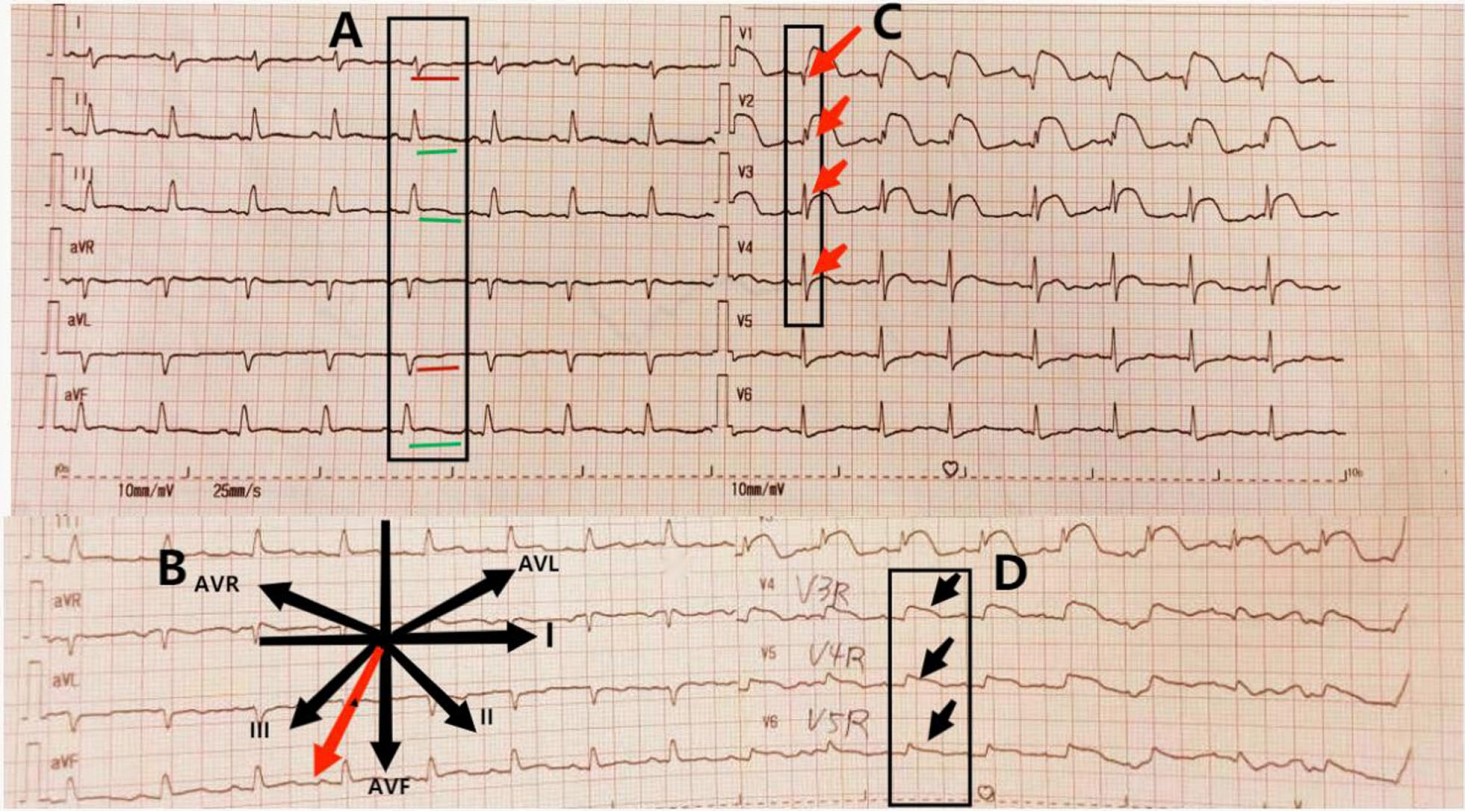
Electrocardiogram (ECG) at presentation: Eighteen-lead electrocardiogram (ECG) showing sinus rhythm with T wave inversion in leads I and AVL, ST-segment elevation in leads V1-V4, II, III, AVF, and V3R-V5R. **A** Green lines indicate ST segment elevation in II, III, AVF in limb leads. Red lines show T-wave inversion in leads I and AVL. **B** The electrical axis is biased towards lead III. **C** Red arrows indicate that the R waves in V1–V4 leads did not increase poorly. **D** Black arrows show ST segment elevation in leads V3R–V5R

**Fig. 2:**
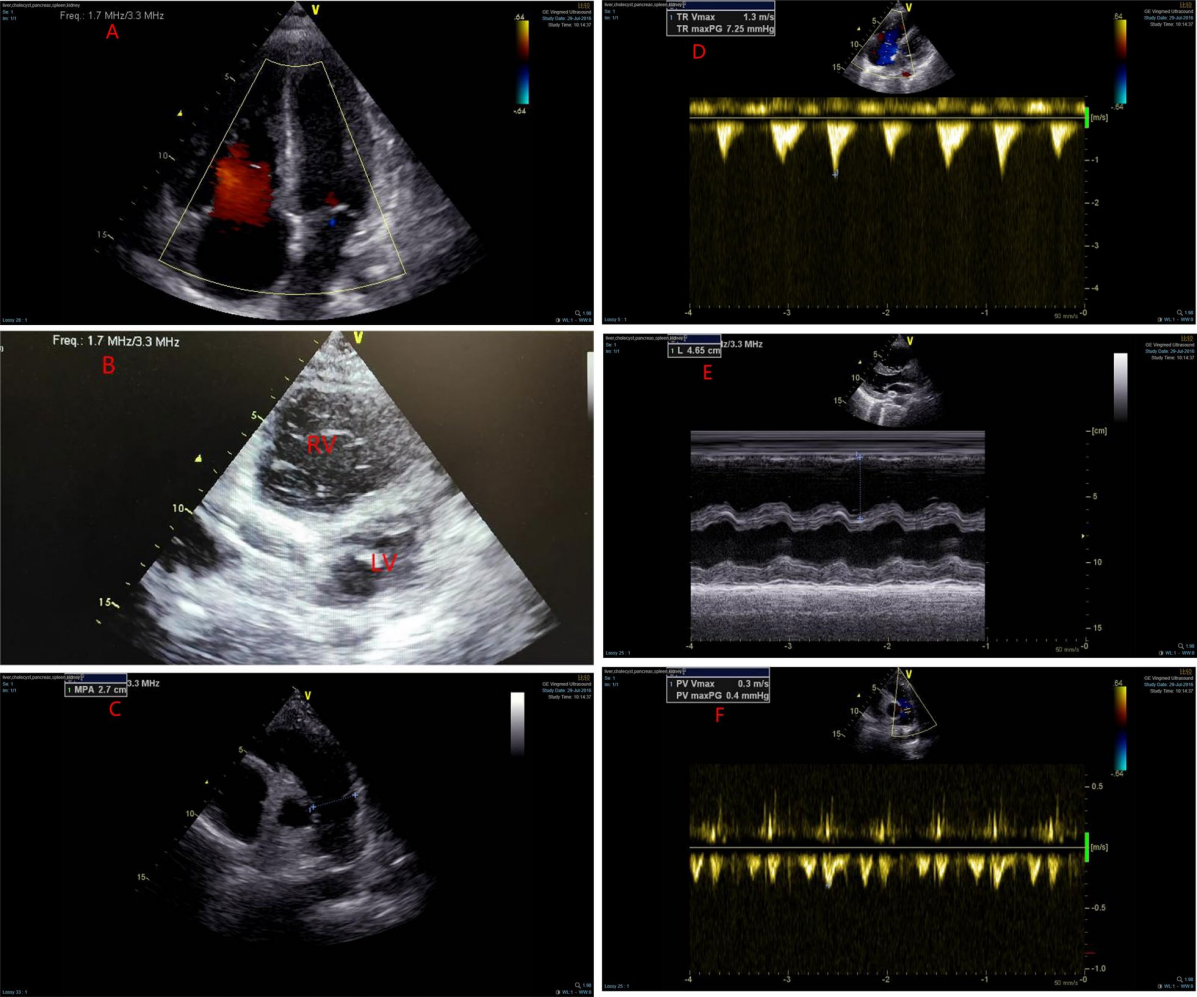
Cardiac Ultrasonographic image at presentation: Ultrasonographic image showing dilated right atrium and ventricle, severe regurgitated tricuspid valve and normal inner diameter of pulmonary artery. **A**, **D** Severe tricuspid valve regurgitation. **B, E** Marked right ventricular dilation. **C**, **F** Normal inner diameter of pulmonary artery

A 37-year-old woman was admitted to our emergency department (ED) with the chief complaint of chest oppression. Past medical history included lumbar disc herniation syndrome, which recurred 20 days ago, accompanied by weakness of limbs and joint pain throughout the body. These symptoms progressively worsened in days. In addition, she developed remarkable anxiety, irritability, and diaphoresis. Approximately 6 h prior to the arrival in ED, the patient developed persistent oppression in chest along with dyspnea. The patient had stable vital signs at admission. Eighteen-lead electrocardiogram (ECG) demonstrated sinus rhythm with T wave inversion in leads I and AVL, ST-segment elevation in leads V1–V4, II, III, AVF and V3R–V5R (Fig. 1). Troponin T level was 23.6 ng/mL (reference range, 0–0.040 ng/mL), D-dimer was 209 ng/mL (reference range, 0–243 ng/mL). The diagnosis of acute coronary syndrome was assumed initially, and an emergency coronary angiography was performed. However, no significant stenosis in the main coronary artery was observed. Urgent transthoracic echocardiogram revealed dilated right atrium and ventricle, severe tricuspid regurgitation, and mild pericardial effusion (Fig. 2). Declined right ventricle systolic function and left ventricle systolic function were observed. The estimated left ventricular ejection fraction (LVEF) was 42.5%. The pulmonary artery pressure was 20 mmHg.

On 1st day after admission, the patient developed atrial flutter with transient hypotension. Subsequently, atrial flutter spontaneously recovered to sinus rhythm. On 2nd day, the patient developed bilateral drooping of eyelids and symmetric flaccid quadriparesis of Medical Research Council (MRC) grade 0/5 in the left upper limb, 2/5 in the right upper limb, and 2/5 in the lower limbs. Cerebrospinal fluid (CSF) was acellular with protein level of 0.77 g/L (reference range, 0.15–0.45 g/L). Nerve conduction studies showed decreased F wave in bilateral peroneal nerves and left median nerve. Both neurophysiological and CSF examinations were consistent with the diagnosis of Guillain–Barré Syndrome. Treatment with intravenous immunoglobulin was started. On 4th day after admission, the patient developed ventricular tachycardia with persistent hypotension. Despite aggressive rescue treatment with electrical cardioversion and vasoactive drugs, the vital signs of the patient continued to worsen until she was ruled dead clinically.

## Discussion

GBS is a polyneuropathy affecting the nervous system characterized by ascending paralysis with weakness beginning in the feet and hands, which typically occurs following an acute infection. In addition, autonomic nerve involvement is a common feature of GBS. Autonomic dysfunction in GBS is typically accompanied with elevated plasma norepinephrine, plasma epinephrine, and 24-h urine Vanillylmandelic acid levels, as well as deranged neurotransmitter metabolism in the CSF [3]. It is now well documented that myocardial dysfunction in GBS can be linked to TC by means of a transient hyperadrenergic state [4].

TC is a reversible myocardial injury triggered by emotional or physical stress [5]. Typical TC involves apical ballooning of the left ventricle during systole with likely hyperkinesis of the basal segments. The atypical type of TC involves the basal, focal, mid-ventricular, biventricular (apical and right ventricle), isolated right ventricular (RV), and global variants. The clinical presentation of TC mimics acute coronary syndrome, including symptoms, electrocardiogram changes, and changes in cardiac biomarker levels; however, TC is distinguished by the absence of significant obstructive coronary artery disease. Coronary angiography is mandatory in such cases to exclude pathological stenosis. The reported prevalence of right ventricular involvement in TC is 25–28% [5], and the incidence of in-hospital complications is higher in such cases. Early identification of the potential mechanical and electrophysiological complications of right ventricular involvement can provide additional information for clinical management and therapy. Echocardiography plays an important role in the first-line diagnosis of isolated RVTC. Several classical ECG features can facilitate the differential diagnosis in patients who have manifestations similar to that of acute coronary syndrome. ECG abnormalities in isolated RVTC are detected mainly in the V1 precordial lead [6]. In some cases, ECG showed QR in V1 lead and transient (visible only in initial tracing) discrete ST elevation in V1–2. Other reported abnormalities in lead V1 are complete or incomplete right bundle branch block or R/S > 1; negative T waves in V1–3 (V2–4) leads and in right precordial leads may also occur. QR in V1 has been also described in other cases of RV strain such as acute pulmonary embolism [7].

**Fig. 3:**
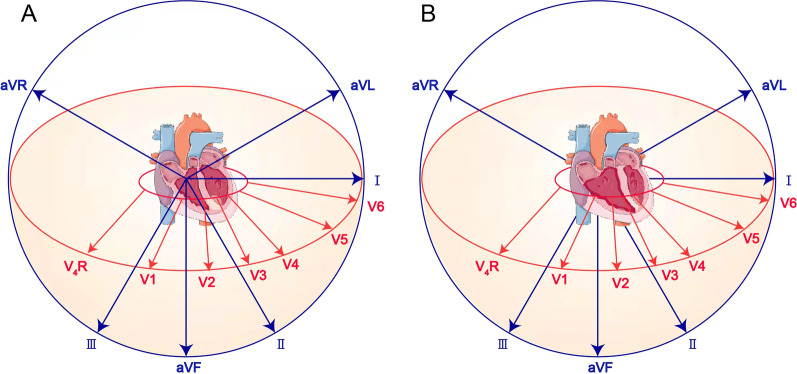
**A** Schematic illustration of the relationship between normal electrical axis, ECG leads, and heart ventricles. **B** V1–V3 leads, and even V4 leads, are closer to the right ventricle, when the right ventricle is enlarged. Therefore, the ST segment elevation in leads V1–V3 is indicative of right ventricular disease

**Fig. 4:**
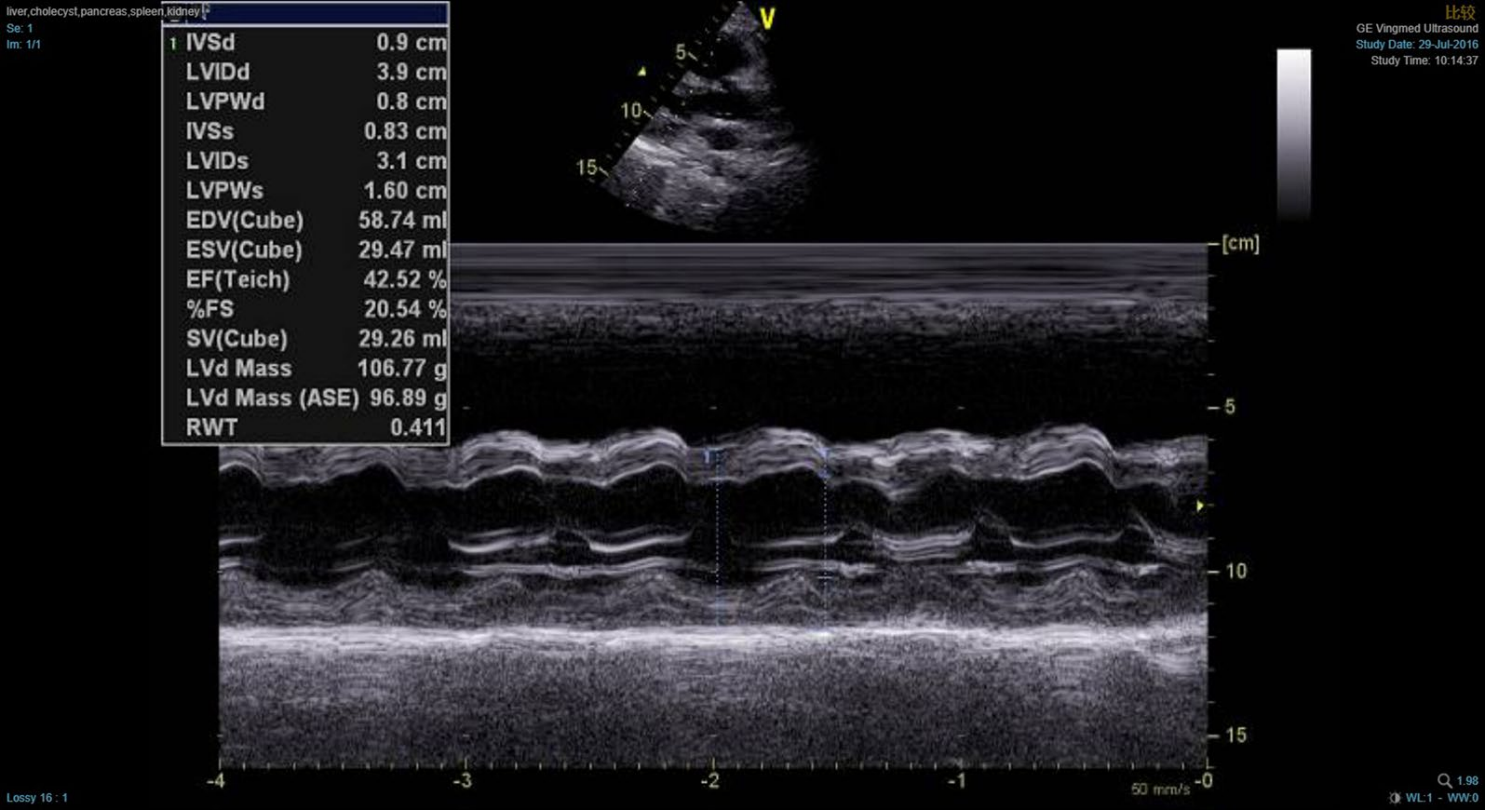
Reduced left ventricular end-diastolic volume (EDV), end-systolic volume (ESV), and stroke volume (SV)

In the present case, ECG limb leads showed ST segment elevation in leads II, III, and AVF which indicated involvement of the under wall of ventricle. From the perspective of vector analysis, the electrical axis should be biased towards lead III, because of T-wave inversion in leads I and AVL. ECG chest leads revealed ST segment elevation dorsally upwards in leads V1–V4 which indicated involvement of the anterior wall of the left ventricle. However, the R waves in V1–V4 leads did not increase poorly, which did not support the involvement of anterior wall of the left ventricle. In the setting of right ventricular enlargement, as in pulmonary embolism, V1–V3 leads, and even V4 leads, are closer to the right ventricle. Therefore, in our case, ST segment elevation in lead V1–V4 and normal interval vector suggested right ventricular involvement (Fig. 3). In addition, lower wall lead elevation, electrical axis biased towards lead III, and ST segment elevation in leads V3R–V5R also pointed towards right ventricular involvement. On the other hand, echocardiogram revealed dilated right ventricle with hypokinesia and severe tricuspid regurgitation (Additional file 1), which is suggestive of isolated right ventricle disease, such as pulmonary embolism. Due to modest elevation of pulmonary artery pressure and negative D dimer, pulmonary embolism was ruled out. Although the systolic function was impaired, the dimensions of left ventricle were normal. By inference, the reduced left ventricular systolic function was caused by the compression of the dilated right ventricle (Additional file 2), leading to lower end-diastolic volume (EDV), end-systolic volume (ESV), stroke volume (SV), dyskinetic left ventricle, and reduced LVEF (Fig. 4). These findings collectively indicate right ventricular involvement in our patient.

The autonomic dysfunction caused by GBS may have acted as a trigger for myocardial damage. According to the Mayo Clinic criteria (2008) [8], our case had new ECG abnormalities and elevated cardiac troponin which could not be explained by regional coronary hypoperfusion, but there was lack of typical left ventricular wall movement abnormalities. However, as mentioned above, the ECG abnormalities in our patient involved the right ventricle. Therefore, we concluded that this GBS patient had isolated right ventricular abnormalities related to TC.

To the best of our knowledge, no previous reports have described isolated RVTC in GBS. We presented a case of GBS who had ECG abnormalities consistent with isolated RVTC which has never been described in literature before. The new ECG presentation linked to isolated RV involvement may prompt early identification of right ventricular involvement in diseases, especially in the setting of an acute ischemic presentation that is not linked to coronary artery disease.

## Supplementary Information

**Additional file 1**. The apical four chamber view. The echocardiogram revealed the dilated right atrium and ventricle.

**Additional file 2**. Short axis view of ventricle. The echocardiogram revealed a D-shaped left ventricle which was caused by the compression of the dilated right ventricle.

## Data Availability

All relevant data supporting the conclusions of this article are included within the article.

## Abbreviations

GBS: Guillain–Barré syndrome
RVTC: Right ventricular Takotsubo cardiomyopathy
ECG: Electrocardiogram
ED: Emergency department
CSF: Cerebrospinal fluid
EDV: End-diastolic volume
ESV: End-systolic volume
SV: Stroke volume
